# Predictors and Prevention of Recurrence After Secondary Spontaneous Pneumothorax: A Systematic Review

**DOI:** 10.7759/cureus.114609

**Published:** 2026-08-16

**Authors:** Mohamed Abdulmajeed, Omar Mahrous, Mohamed Eldahrawy, Moemen Hasaballah, Khaled Elmagraby

**Affiliations:** 1 General Internal Medicine, Milton Keynes University Hospital, Milton Keynes, GBR; 2 General Internal Medicine, Ysbyty Gwynedd Hospital, Bangor, GBR; 3 Internal Medicine, Lagan Valley Hospital, Lisburn, GBR; 4 General Internal Medicine, Luton and Dunstable Hospital, Luton, GBR; 5 Acute Internal Medicine, Milton Keynes University Hospital, Milton Keynes, GBR

**Keywords:** persistent air leak, pneumothorax recurrence, recurrence predictors, secondary spontaneous pneumothorax, video-assisted thoracic surgery

## Abstract

Secondary spontaneous pneumothorax (SSP) carries a clinically important risk of recurrence, but the available evidence is dispersed across heterogeneous disease-specific and treatment cohorts. This systematic review aimed to identify clinical, radiological, physiological, disease-related, clinical-course, and treatment-related determinants of recurrence after SSP.

PubMed/MEDLINE was searched on 19 July 2026 without a date restriction. Adult and adult-inclusive SSP cohorts were eligible, as were mixed spontaneous-pneumothorax cohorts containing patients with SSP when a recurrence determinant was evaluated. Evidence without SSP-specific estimates was classified as indirect. Of 77 records screened, 50 reports were sought, 31 were retrieved and assessed, two were excluded at full text, and 29 studies were included.

Nineteen reports could not be retrieved and were classified as unavailable rather than scientifically excluded. Because of substantial heterogeneity in populations, interventions, recurrence definitions, follow-up periods, and effect measures, a narrative synthesis was undertaken without meta-analysis. Recurrence was associated with severe emphysematous or fibrotic lung damage, non-chronic obstructive pulmonary disease (non-COPD) aetiology, prolonged air leak, impaired forced expiratory volume in one second (FEV1), Birt-Hogg-Dubé syndrome, chest deformity in Marfan syndrome, and selected malignancy-related radiographic features.

Definitive recurrence prophylaxis, video-assisted thoracoscopic surgery (VATS)-based procedures, pleural interventions, and medical pleurodesis were generally associated with lower recurrence than drainage alone. Technical comparisons suggested that the effectiveness of lesion control and pleural intervention may be more important than the number of thoracoscopic ports. Evidence favouring thoracotomy over VATS arose from mixed cohorts and should be interpreted cautiously. SSP recurrence risk should therefore be assessed using the underlying disease, structural lung damage, physiological reserve, behaviour of the index episode, and suitability for definitive pleural intervention.

## Introduction and background

Secondary spontaneous pneumothorax (SSP) occurs in patients with clinically apparent underlying pulmonary or systemic disease and is often complicated by limited cardiopulmonary reserve and substantial management complexity. The underlying disease phenotype has an important influence on presentation, treatment requirements, recurrence, and longer-term outcomes. Available disease-specific evidence includes cohorts of patients with chronic pulmonary disease, non-chronic obstructive pulmonary disease (non-COPD) pathology, malignancy-associated pneumothorax, and heritable connective-tissue disorders, demonstrating that SSP is a heterogeneous clinical entity rather than a single uniform condition [1-4].

Recurrence following SSP is clinically important because another episode may result in repeat hospitalisation, prolonged pleural drainage, further pleural intervention, surgery, and deterioration in respiratory function. Reported recurrence determinants include the severity of emphysematous or fibrotic structural lung damage, the underlying pulmonary diagnosis, persistent air leak, and the clinical behaviour of the index episode. Observational studies have particularly identified severe computed tomography (CT)-defined emphysema, non-COPD pathology, and an air leak persisting for more than seven days as potentially important recurrence markers [2,5,6].

Treatment-related evidence suggests that definitive management of the responsible pulmonary lesion and pleural space may reduce recurrence compared with drainage alone. Same-admission recurrence prophylaxis has been associated with lower short- and longer-term readmission for recurrent SSP, while video-assisted thoracoscopic surgery (VATS) with bullectomy and partial pleurectomy has demonstrated lower recurrence than chest-tube drainage in selected patients [7,8]. However, these findings arise predominantly from retrospective observational studies and remain susceptible to treatment-selection bias and confounding by indication.

The available literature is heterogeneous in relation to the underlying disease, study population, intervention, recurrence definition, laterality, follow-up duration, and reported effect measure. This systematic review therefore aimed to identify and synthesise clinical, radiological, physiological, disease-related, clinical-course, and treatment-related determinants of recurrence after SSP and to distinguish direct SSP evidence from indirect evidence derived from mixed spontaneous-pneumothorax cohorts.

## Review

Materials and methods 

Design and Reporting

This systematic review was conducted and reported in accordance with the Preferred Reporting Items for Systematic Reviews and Meta-Analyses 2020 statement (PRISMA 2020) and relevant PRISMA-S recommendations for reporting literature searches [9,10]. The review evaluated studies published from database inception to 19 July 2026. A narrative synthesis was planned because substantial heterogeneity was anticipated in the underlying diseases, study populations, interventions, recurrence definitions, follow-up periods, and reported effect measures. The completed PRISMA 2020 checklist is provided in the Appendices section. The review protocol was not prospectively registered with PROSPERO or another systematic-review registry.

Information Source and Search Strategy

PubMed/MEDLINE was searched from database inception to 19 July 2026, with no lower date restriction. PubMed/MEDLINE was selected as the sole bibliographic database because the review addressed a focused clinical question within respiratory and thoracic medicine and because it provides broad, consistently indexed biomedical coverage through a reproducible search interface. The authors recognise that a single-database strategy may have missed relevant studies indexed only in other bibliographic databases; accordingly, this review should be interpreted as a focused PubMed-based synthesis rather than an exhaustive search of all available literature.

The search combined terms for SSP or underlying lung disease with terms relating to recurrence, prognostic factors, surgery, pleurodesis, and persistent air leak. Boolean operators AND, OR, and NOT were used to combine the concepts. The applied PubMed filters were English language, humans, and adults. The principal search structure was: (“secondary spontaneous pneumothorax” OR SSP OR [pneumothorax AND underlying-lung-disease terms]) AND (recurren* OR recurrent OR relapse OR “recurrence-free”) AND (predict* OR prognos* OR “risk factor*” OR determinant* OR pleurodesis OR surgery OR thoracoscop* OR VATS OR bullectom* OR “persistent air leak”) NOT (traumatic OR iatrogenic OR paediatric, neonatal, or catamenial terms).

The complete reproducible PubMed/MEDLINE search string, including all field tags, Medical Subject Headings, Boolean operators, and exclusion terms, is presented in Appendix 1.

Eligibility Criteria

Full-text original observational studies were eligible when they met the following criteria: (i) included adults with SSP, an adult-inclusive disease-specific SSP cohort, or a mixed spontaneous-pneumothorax cohort containing patients with SSP; (ii) reported pneumothorax recurrence, recurrence-free survival, time to recurrence, recurrence-related readmission, or recurrence-related reoperation; and (iii) evaluated at least one clinical, radiological, physiological, disease-related, clinical-course, or treatment-related determinant of recurrence.

Direct evidence was defined as SSP-only cohorts or studies providing separately extractable SSP findings. Adult-inclusive disease cohorts were retained when they were not exclusively paediatric. Mixed spontaneous-pneumothorax studies were included when SSP was represented and a relevant recurrence determinant was evaluated; findings without SSP-specific estimates were classified as indirect evidence and were not interpreted as direct SSP effect estimates.

Primary spontaneous pneumothorax (PSP)-only studies, traumatic or iatrogenic pneumothorax studies, exclusively paediatric or neonatal cohorts, catamenial pneumothorax studies, case reports, reviews, editorials, non-English publications, and studies without an eligible post-index recurrence outcome or recurrence determinant were excluded. Reports that could not be obtained were recorded as not retrieved rather than excluded on scientific grounds.

Study Selection and Data Extraction

Two reviewers independently screened the titles and abstracts against the prespecified eligibility criteria. Potentially eligible reports were then assessed independently at full text. Disagreements were resolved through discussion and consensus, with involvement of a third reviewer when required.

Full-text retrieval was attempted through PubMed and PubMed Central links, DOI searches, publisher websites, and available NHS or university library access. Where a report remained unavailable and corresponding-author contact details could be identified, the authors were contacted to request a copy. Reports that remained unobtainable after these attempts were recorded as “reports not retrieved” rather than excluded on scientific grounds.

Two reviewers independently extracted data using a structured form. Extracted variables included study design, country and setting, sample size, patient characteristics, underlying disease, SSP definition, recurrence definition, follow-up period, investigated predictor or intervention, recurrence frequency, effect estimates, confidence intervals, statistical significance, and directness of the evidence. Extracted findings were checked against the corresponding full-text reports, and discrepancies were resolved by consensus. Reports initially classified as uncertain were reassessed using the same prespecified eligibility criteria.

Risk-of-Bias Assessment

Two reviewers independently assessed the included observational studies using the Newcastle-Ottawa Scale (NOS) [11]. The NOS evaluates three domains: selection of the study population, comparability of the study groups, and ascertainment of outcomes. The maximum possible scores were four points for selection, two points for comparability, and three points for outcome assessment, giving a maximum total score of nine. Higher scores were interpreted as indicating lower risk of bias.

Differences in scoring were resolved through discussion and consensus, with a third reviewer consulted when required. Mixed-cohort studies were additionally interpreted cautiously when SSP-specific estimates were unavailable or when recurrence was incorporated within a composite readmission or reoperation outcome. Individual NOS domain and total scores are presented in the Results section.

Data Synthesis

The findings were synthesised narratively and grouped into structural and disease-related predictors, physiological and clinical-course predictors, treatment-related determinants, and special populations. Study characteristics, patient populations, predictors, interventions, recurrence outcomes, and effect estimates were summarised in tables and accompanying text.

Quantitative findings, including recurrence proportions, hazard ratios, odds ratios, adjusted odds ratios, confidence intervals, and p-values, were reported as presented by the original studies. A quantitative meta-analysis was not performed because of substantial clinical and methodological heterogeneity in the underlying diseases, patient selection, interventions, recurrence definitions, laterality of recurrence, follow-up periods, and effect measures. Consequently, no pooled effect estimate or formal assessment of statistical heterogeneity was calculated. Following application of the prespecified eligibility criteria, 29 studies were included in the final qualitative synthesis; the study-selection process is detailed in the Results section and PRISMA 2020 flow diagram.

Results 

Study Selection 

**Figure 1:**
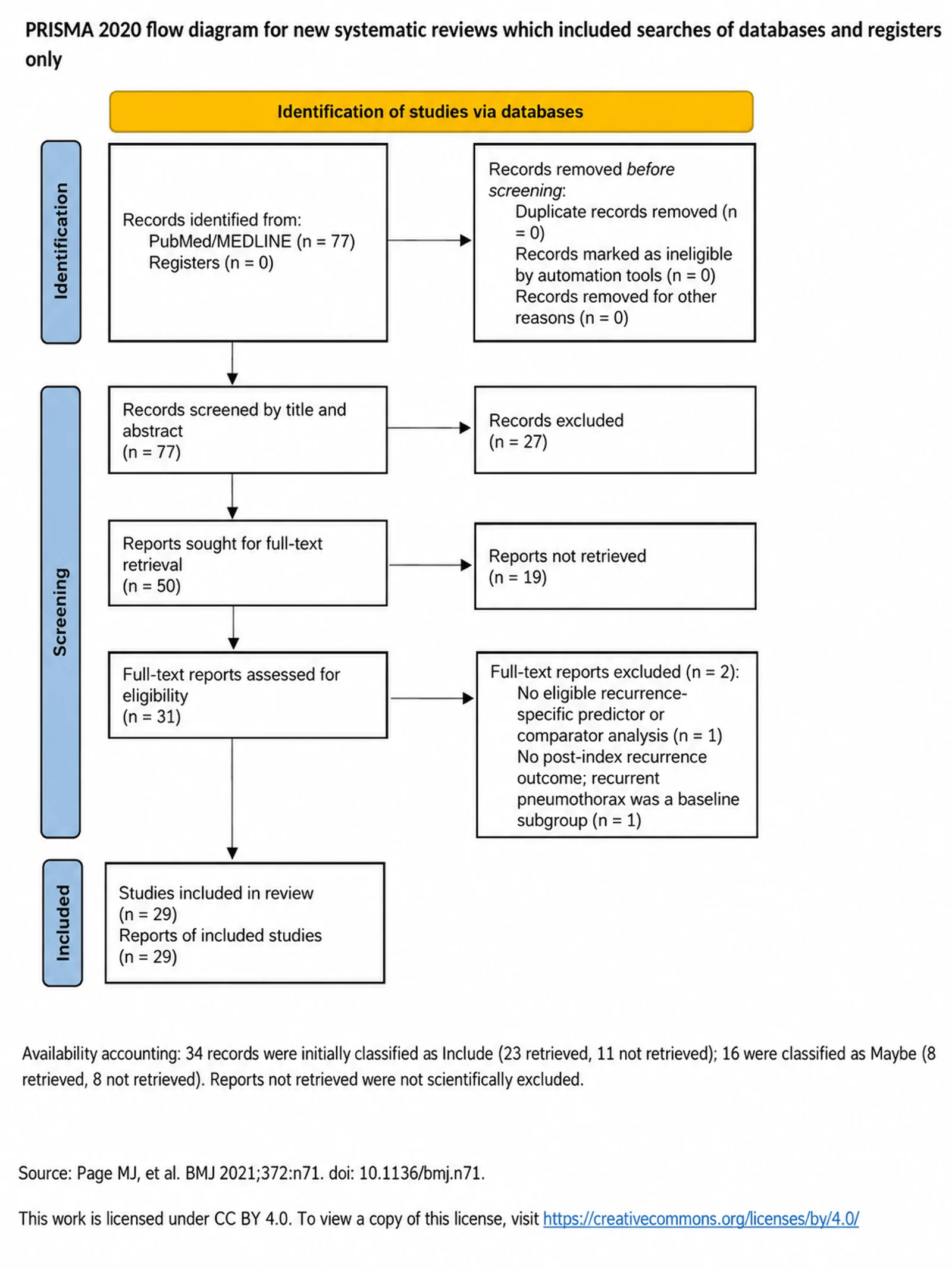
PRISMA 2020 flow diagram The figure was created using the official PRISMA 2020 flow-diagram template and adapted by the authors using the study-selection data from this review. Source: Page et al. [9].

The search yielded 77 records, all of which underwent title and abstract screening. Twenty-seven records were excluded, and 50 reports were sought for full-text retrieval. Thirty-one full-text reports were successfully retrieved and assessed, including eight of the 16 records initially classified as uncertain. Full-text retrieval was attempted through PubMed and PubMed Central links, DOI searches, publisher websites, NHS or university library access, and contact with corresponding authors where contact details were available. Nineteen reports remained unavailable: 11 of the 34 records initially classified as “Include” and eight of the 16 records classified as “Maybe.” These reports were retained in the PRISMA category “reports not retrieved” rather than excluded on eligibility grounds. Detailed retrieval and availability accounting is presented in Appendix 2. Two reports were excluded after full-text assessment: one reported postoperative recurrence but did not evaluate an eligible recurrence-specific predictor or comparator, and the other used recurrent pneumothorax as a baseline subgroup without evaluating a post-index recurrence outcome or determinant of subsequent recurrence. The excluded reports and corresponding reasons are presented in Appendix 3. Twenty-nine studies were included in the qualitative synthesis (Figure 1).

Study Characteristics

The 29 included studies were published between 2006 and 2026 and were predominantly retrospective observational studies, including disease-specific medical cohorts, surgical cohorts, nationwide admission or readmission databases, and mixed spontaneous-pneumothorax cohorts. Sample sizes ranged from 14 pneumothorax cases within an adult-inclusive disease-specific cohort to 21,838 SSP admissions. The study populations included patients with COPD or emphysema, interstitial lung disease or pulmonary fibrosis, malignancy-associated SSP, infectious lung disease, persistent air leak, and heritable disorders including Birt-Hogg-Dubé and Marfan syndromes. Twenty studies provided direct SSP or separately extractable SSP evidence, two were adult-inclusive disease cohorts, and seven provided mixed-cohort, indirect, or composite-outcome evidence requiring cautious interpretation [1-8,12-32].

Across the included studies, recurrence was most consistently associated with severe CT-defined emphysema, fibrotic or non-COPD lung disease, impaired post-recovery forced expiratory volume in one second (FEV1), persistent air leak lasting more than seven days, early previous recurrence, Birt-Hogg-Dubé syndrome, chest deformity in Marfan syndrome, and selected malignancy-related radiographic features [2-6,12-15,25]. Reported recurrence varied according to the underlying disease and treatment strategy. Same-admission recurrence prophylaxis was associated with lower recurrence at 30 days, 90 days, and one year [7]. Recurrence was 16.7% following VATS bullectomy with partial pleurectomy compared with 41.3% after chest-tube treatment [8], and 4.5% following VATS with talc pleurodesis compared with 30% after conventional treatment [26]. Evidence comparing individual surgical techniques and pleurodesis strategies was more heterogeneous and was frequently based on small or indirect cohorts [21-24,27-32]. The characteristics and recurrence findings of included studies are provided in Table 1. Individual NOS domain and total scores are presented in Table 2.

**Table 1:** Characteristics and recurrence findings of included studies Direct SSP evidence represents SSP-only cohorts or separable SSP subgroups. Adult-inclusive evidence includes adolescents and adults. Mixed or indirect evidence includes studies in which SSP-specific recurrence estimates were unavailable or the outcome was composite. SSP, secondary spontaneous pneumothorax; PSP, primary spontaneous pneumothorax; SP, spontaneous pneumothorax; COPD, chronic obstructive pulmonary disease; ILD, interstitial lung disease; NTM, nontuberculous mycobacteria; CT, computed tomography; FEV1, forced expiratory volume in one second; VATS, video-assisted thoracoscopic surgery; SEC, staphylococcal enterotoxin C; HR, hazard ratio; OR, odds ratio; aOR, adjusted odds ratio.

Ref	Study, year, country	Design and sample	Population	Predictor or exposure	Main recurrence finding	Evidence classification
[1]	Cheng et al. (2026), Hong Kong	Retrospective cohort; n=223	SSP across acute infection and chronic lung disease	Underlying disease phenotype	Two-year recurrence varied by disease; one-year recurrence was 8.3% in asthma and 0% in bronchiectasis versus 15.8%-33.3% in other groups.	Direct SSP
[2]	Moon et al. (2023), South Korea	Retrospective surgical cohort; n=160	First surgery for secondary pneumothorax	Underlying disease	Overall recurrence 18.75%; non-COPD pathology predicted recurrence (HR 5.3, p<0.001).	Direct SSP
[3]	Grosu et al. (2019), USA	Retrospective competing-risk cohort; n=96 evaluable	Malignancy-associated SSP	Cancer type and radiographic dimensions	Recurrence 9.4%; mediastinal shift (HR 12.30), larger pneumothorax dimensions, and sarcoma were associated with recurrence.	Direct SSP
[4]	Kutsukake et al. (2024), Japan	Nationwide cohort; 524 patients, 884 admissions	Heritable connective-tissue disorder-associated SSP	Age, syndrome, surgery	Surgery reduced readmission (HR 0.47); younger age (HR 2.46) and Birt-Hogg-Dube syndrome (HR 2.53) increased recurrence.	Direct SSP
[5]	Isaka et al. (2013), Japan	Retrospective surgical cohort; 94 patients, 97 operations	Emphysematous SSP	CT emphysema score, fibrosis, technique	Recurrence 9.3%; Goddard score >=7 predicted recurrence (HR 7.79); fibrosis showed a strong trend (HR 4.21).	Direct SSP
[6]	Cheng et al. (2023), Hong Kong	Retrospective non-surgical cohort; n=180	Chest-drain-treated SSP	Persistent air leak and pleurodesis	Air leak >7 days predicted recurrence at 3 months, 1 year, and 2 years (HR 2.40-2.65); medical pleurodesis was protective at 2 years (HR 0.47).	Direct SSP
[7]	Wang et al. (2020), USA	Nationwide readmission cohort; 21,838 admissions	Adult SSP admissions	Same-admission recurrence prophylaxis	Prophylaxis reduced recurrence at 30 days (aOR 0.22), 90 days (aOR 0.26), and 1 year (aOR 0.22).	Direct SSP
[8]	Fung et al. (2022), Germany	Retrospective comparative cohort; n=82	SSP treated by surgery or chest tube	VATS bullectomy + partial pleurectomy vs chest tube	Recurrence 16.7% after surgery versus 41.3% after chest tube (p=0.016).	Direct SSP
[12]	Chang et al. (2016), Taiwan	Retrospective surgical cohort; 84 patients, 96 operations	Emphysema-related SSP	Goddard score; pleural tenting	Goddard score predicted recurrence (OR 2.525, p=0.019); pleural tenting reduced secondary procedures.	Direct SSP
[13]	Shin et al. (2020), South Korea	Retrospective cohort; 49 analysed	First SSP with CT-defined emphysema	Post-recovery pulmonary function	Recurrence was 30.6%. Higher post-recovery FEV1 was associated with a lower recurrence risk (adjusted HR 0.408, p=0.025), indicating that lower FEV1 predicted recurrence.	Direct SSP
[14]	Ichinose et al. (2016), Japan	Retrospective surgical cohort; n=183	Surgically treated SSP	Underlying disease and approach	Interstitial pneumonia was associated with higher recurrence and treatment failure.	Direct SSP
[15]	Yanagiya et al. (2025), Japan	Retrospective surgical cohort; n=20	Marfan-associated secondary pneumothorax; age 13-40 years	Chest deformity	Cumulative postoperative pneumothorax was 25% at 5 years and 44% at 10 years; chest deformity predicted recurrence (HR 7.63).	Adult-inclusive disease cohort
[16]	Tabur et al. (2025), Turkiye	Retrospective cohort; 2,797 episodes	Adult traumatic and spontaneous pneumothorax	Age, SSP, smoking, COPD	Age >=65 (OR 1.92), SSP (OR 1.88), smoking (OR 1.51), and COPD (OR 1.49) independently predicted recurrence.	Mixed cohort; SSP as predictor
[17]	Farag et al. (2024), Egypt	Retrospective mixed cohort; n=170, 57.1% SSP	Adult spontaneous pneumothorax	Age, smoking, blebs/bullae, blood indices, drainage	SSP had higher recurrence than PSP; older age, blebs/bullae, current smoking, lower haemoglobin/haematocrit, and longer drainage were associated with recurrence overall.	Mixed cohort with SSP subgroup
[18]	Stefani et al. (2020), Italy	Retrospective case-control surgical cohort; n=112	Bullous SP stratified by cannabis/tobacco exposure	Cannabis and tobacco smoking	Postoperative recurrence was more frequent among cannabis smokers; 7 of 39 cannabis smokers recurred.	Mixed cohort; secondary bullous disease
[19]	Ueyama et al. (2016), Japan	Retrospective multicentre cohort; n=69	NTM pulmonary disease-associated SSP	Clinical/radiological factors and treatment	Eighteen patients recurred; recurrence-free proportion was 59% at 3 years. Adjusted analysis mainly addressed mortality.	Direct SSP
[20]	Lee et al. (2016), South Korea	Retrospective cohort; n=216	SSP in adults >70 years	Old pulmonary tuberculosis	Old tuberculosis prolonged drainage and complications but did not significantly alter recurrence-free survival.	Direct SSP
[21]	Akar et al. (2020), Turkiye	Retrospective comparative cohort; n=42	SSP with persistent air leak >7 days	60 vs 120 mL autologous blood patch	Recurrence was compared; the 120-mL group had better overall outcomes, but no adjusted recurrence estimate was reported.	Direct SSP
[22]	Evman et al. (2016), Turkiye	Retrospective cohort; n=31	First-episode SSP with resistant air leak	Autologous blood-patch pleurodesis	Late recurrence occurred in 4 patients (12.9%).	Direct SSP
[23]	Aihara et al. (2011), Japan	Retrospective cohort; 34 patients, 59 episodes	ILD-associated SSP	Blood-patch vs chemical pleurodesis	Cure and recurrence ratios were comparable between blood and chemical pleurodesis (p=0.99).	Direct SSP
[24]	Yamagishi et al. (2026), Japan	Retrospective surgical cohort; n=21	High-risk intractable SSP	Non-intubated VATS with postoperative strategy	Postoperative recurrence occurred in 2 patients (11%).	Direct SSP; small cohort
[25]	Kim et al. (2020), South Korea	Retrospective cohort; 99 recurrent SSP cases	Recurrent SSP after initial conservative treatment	Surgery and recurrence-free interval	Surgery prolonged recurrence-free interval after early recurrence (p=0.007), but not when the prior interval exceeded 1 year (p=0.748).	Direct SSP
[26]	Kim et al. (2011), South Korea	Retrospective comparative cohort; n=61	SSP requiring conventional treatment or VATS	VATS + talc vs conventional treatment	Recurrence 4.5% after VATS versus 30% after conventional treatment (p=0.016).	Direct SSP
[27]	Dadaş et al. (2015), Turkiye	Retrospective surgical cohort; 67 patients, 72 procedures; 15 SSP	Mixed SP with SSP subgroup	Total pleurectomy, apical pleurectomy + abrasion, or non-pleurectomy pleurodesis	All 3 recurrences occurred in SSP; within SSP, recurrence was 0/3, 1/7, and 2/7, respectively.	SSP subgroup; very small cells
[28]	Janssen et al. (2024), Netherlands	Retrospective cohort; n=212, 133 SSP	Surgically treated mixed SP	Uniportal vs multiportal VATS	Recurrence was 6% versus 6%; surgical approach was not an independent predictor.	Indirect mixed-cohort evidence
[29]	Ingolfsson et al. (2006), Sweden	Retrospective cohort; 240 patients, 256 operations; 67 SSP	Operated mixed SP	Patient factors and operative technique	Secondary pneumothorax due to emphysema predicted reoperation (OR 3.62); wedge resection was protective (OR 0.23). Outcome combined early and late reoperation.	Indirect/composite outcome
[30]	Delpy et al. (2016), France	National surgical database; n=7,647	Primary or secondary SP; subtype not separable	VATS vs thoracotomy; resection and pleurodesis	Recurrence was 3.8% after VATS versus 1.8% after thoracotomy; thoracotomy (OR 0.40) and bullectomy (OR 0.60) were protective.	Indirect; SSP not separable
[31]	Tian et al. (2021), China	Multicentre retrospective cohort; 54 treated, 14 pneumothorax	Apatinib-associated secondary pneumothorax in osteosarcoma	Chest tube + SEC perfusion vs chest tube alone	Recurrence was 25.0% versus 66.7%, respectively (p=0.277); the comparison was underpowered.	Adult-inclusive; small subgroup
[32]	Fung et al. (2022), Germany	Retrospective surgical cohort; n=115	SSP undergoing VATS	Two-port vs three-port VATS	Long-term recurrence was similar between approaches.	Direct SSP

**Table 2:** Newcastle-Ottawa Scale appraisal Maximum scores were four points for selection, two points for comparability, and three points for outcome, giving a maximum total score of nine. Higher scores indicate a lower risk of bias. Citation numbers correspond to the complete references in the reference list and to the studies presented in Table 1. NOS, Newcastle-Ottawa Scale; S, selection; C, comparability; O, outcome.

Study	S	C	O	Total	Study	S	C	O	Total
Cheng et al. (2026) [1]	4	1	2	7	Ueyama et al. (2016) [19]	4	1	2	7
Moon et al. (2023) [2]	4	1	2	7	Lee et al. (2016) [20]	4	1	2	7
Grosu et al. (2019) [3]	4	2	2	8	Akar et al. (2020) [21]	3	1	2	6
Kutsukake et al. (2024) [4]	4	2	2	8	Evman et al. (2016) [22]	3	1	2	6
Isaka et al. (2013) [5]	4	1	2	7	Aihara et al. (2011) [23]	3	1	2	6
Cheng et al. (2023) [6]	4	2	2	8	Yamagishi et al. (2026) [24]	3	1	2	6
Wang et al. (2020) [7]	4	2	2	8	Kim et al. (2020) [25]	4	1	2	7
Fung et al. (2022) (VBPP) [8]	4	1	2	7	Kim et al. (2011) [26]	3	1	2	6
Chang et al. (2016) [12]	4	1	2	7	Dadas et al. (2015) [27]	3	1	2	6
Shin et al. (2020) [13]	4	1	2	7	Janssen et al. (2024) [28]	4	1	2	7
Ichinose et al. (2016) [14]	4	1	2	7	Ingolfsson et al. (2006) [29]	4	1	2	7
Yanagiya et al. (2025) [15]	3	1	2	6	Delpy et al. (2016) [30]	3	2	2	7
Tabur et al. (2025) [16]	4	2	2	8	Tian et al. (2021) [31]	3	0	2	5
Farag et al. (2024) [17]	3	1	2	6	Fung et al. (2022) (ports) [32]	4	1	2	7
Stefani et al. (2020) [18]	4	1	2	7	-	-	-	-	-

Structural and Disease-Related Predictors 

Greater structural lung damage was the most reproducible risk domain. In emphysema-related SSP, CT emphysema severity assessed using the Goddard score predicted recurrence: a score of at least seven carried an HR of 7.79 in one surgical cohort, while increasing Goddard score was independently associated with recurrence in another cohort [5,12]. Higher post-recovery FEV1 was associated with a lower recurrence risk in emphysema, indicating that lower FEV1 was a risk marker [13]. Interstitial pneumonia and fibrotic disease were associated with treatment failure, recurrence, or a strong trend toward recurrence, and non-COPD pathology was the strongest predictor after initial secondary-pneumothorax surgery [2,14]. 

Heritable and chest-wall disorders introduced additional risk. Younger age and Birt-Hogg-Dube syndrome increased pneumothorax readmission in a nationwide heritable-connective-tissue-disorder cohort [4]. In Marfan syndrome, the cumulative incidence of postoperative pneumothorax was 25% at five years and 44% at 10 years; chest deformity independently predicted recurrence (HR 7.63, 95% CI 1.29-45.1) [15]. This estimate was imprecise and arose from an adult-inclusive cohort of 20 patients. 

In a large adult emergency cohort, age at least 65 years, SSP status, smoking, and COPD were independent recurrence predictors, although the model combined spontaneous-pneumothorax subtypes [16]. A further mixed cohort reported higher recurrence in SSP and associations with older age, blebs or bullae, current smoking, lower haemoglobin or haematocrit, and longer drainage [17]. Cannabis exposure was associated with more severe bullous disease and more postoperative recurrence in a surgical case-control cohort [18]. 

Malignancy-associated SSP demonstrated a distinct radiographic and disease profile. Mediastinal shift, larger apex-to-cupola distance, greater hilar pleural separation, and sarcoma were associated with recurrence [3]. These predictors may reflect both pneumothorax size and the biology of metastatic disease rather than a universal SSP mechanism. 

Physiological and Clinical-Course Predictors 

Persistent air leak was a clinically useful dynamic marker. In a 180-patient non-surgical SSP cohort, air leak lasting more than seven days predicted recurrence at three months, one year, and two years, with HRs from 2.40 to 2.65 [6]. Persistent leak likely integrates the severity of structural disease, the size or persistence of the pleural defect, and failure of spontaneous pleural healing. 

Recurrence timing also modified treatment effect. Surgery prolonged the recurrence-free interval after early recurrent SSP, but not clearly when the preceding recurrence-free interval exceeded one year [25]. Nontuberculous mycobacterial disease was associated with frequent recurrence and poor survival, although adjusted modelling primarily addressed mortality [19]. Old pulmonary tuberculosis prolonged drainage and complications in patients older than 70 years but did not significantly alter recurrence-free survival [20]. 

Treatment-Related Determinants 

Definitive recurrence prophylaxis generally performed better than drainage alone. Same-admission recurrence prophylaxis was associated with markedly lower recurrence at 30 days, 90 days, and one year in 21,838 adult SSP admissions [7]. VATS bullectomy with partial pleurectomy produced recurrence of 16.7% compared with 41.3% after chest-tube treatment [8], while VATS with talc pleurodesis produced recurrence of 4.5% compared with 30% after conventional treatment [26]. 

The newly retrieved surgical studies refined the technical evidence. In a small SSP subgroup, all three recurrences occurred after less extensive pleural procedures: none after total pleurectomy, one after apical pleurectomy plus abrasion, and two after non-pleurectomy pleurodesis [27]. Uniportal and multiportal VATS had identical crude recurrence rates of 6%, and the approach was not an independent predictor in a cohort containing 133 SSP patients [28]. In another mixed surgical cohort, secondary pneumothorax due to emphysema predicted reoperation (OR 3.62), while wedge resection was protective (OR 0.23); however, the outcome combined early complications and late recurrent pneumothorax [29]. 

A national mixed-SP surgical database reported recurrence of 3.8% after VATS and 1.8% after thoracotomy; thoracotomy and bullectomy were independently protective [30]. Because the database could not separate PSP from SSP, this finding is indirect and should not be used alone to select thoracotomy for SSP. The less invasive benefits of VATS and the extent and quality of pleural intervention remain clinically important. 

Pleurodesis evidence was heterogeneous. In apatinib-associated secondary pneumothorax in osteosarcoma, chest-tube drainage plus staphylococcal enterotoxin C perfusion had a lower recurrence proportion than drainage alone (25.0% vs 66.7%), but the difference was not statistically significant, and the subgroup was very small [31]. Autologous blood-patch studies reported late recurrence of approximately 13% or recurrence comparable with chemical pleurodesis; a larger blood volume improved overall clinical outcomes without a recurrence-specific adjusted estimate [21-23]. High-risk non-intubated VATS was followed by 11% postoperative recurrence in a 21-patient series [24]. Two-port and three-port VATS showed similar long-term recurrence, suggesting that port number is less influential than successful control of the leak and an effective pleural procedure [32]. 

Risk of Bias 

NOS totals ranged from five to eight of nine. Most cohorts had clear case definitions and adequate outcome ascertainment. Comparability was limited by retrospective treatment selection, small samples, incomplete adjustment, referral bias, competing mortality, and inconsistent recurrence definitions. The lowest score was assigned to the small osteosarcoma treatment subgroup because of limited comparability and imprecision. Mixed-SP studies were not treated as equivalent to direct SSP cohorts, even when their NOS scores were otherwise acceptable.

Discussion

This systematic review indicates that recurrence after SSP is not explained by a single universal predictor. Instead, risk appears to arise from the interaction between the underlying disease, the severity of structural lung damage, physiological reserve, the clinical behaviour of the index episode, and whether effective recurrence prophylaxis is performed. Recurrence varied substantially across underlying lung diseases, supporting the view that SSP is a heterogeneous clinical entity rather than one uniform disorder [1]. Non-COPD pathology was strongly associated with recurrence after initial surgery [2], while malignancy-associated SSP demonstrated a distinct profile involving sarcoma and radiographic markers of pneumothorax severity [3]. In heritable connective-tissue disorders, younger age and Birt-Hogg-Dubé syndrome were associated with increased pneumothorax readmission [4]. These findings suggest that recurrence assessment should begin with the underlying disease phenotype rather than pneumothorax size alone.

Structural lung damage was one of the most consistent recurrence domains. In emphysema-related SSP, severe CT-defined emphysema was strongly associated with recurrence, while pulmonary fibrosis showed a clinically important trend [5]. Persistent air leak was another useful predictor because it is directly observable during the index admission. An air leak lasting longer than seven days predicted recurrence at three months, one year, and two years, whereas medical pleurodesis was associated with a lower two-year recurrence risk [6]. Persistent leakage may therefore represent an integrated marker of extensive parenchymal damage, a continuing visceral pleural defect, and failure of spontaneous pleural healing.

Treatment-related evidence generally favoured active recurrence prevention over drainage alone. Same-admission recurrence prophylaxis was associated with substantially lower recurrence at 30 days, 90 days, and one year in a nationwide cohort of adult SSP admissions [7]. Similarly, VATS with bullectomy and partial pleurectomy resulted in lower recurrence than chest-tube treatment alone [8]. Although these studies were observational and susceptible to confounding by indication, the direction of effect was consistent: successful resolution of the initial episode without treating the responsible lesion or pleural space may leave a clinically important risk of recurrence.

Additional evidence supported the prognostic value of radiological severity and physiological reserve. Severe CT emphysema was independently associated with recurrence in another surgical cohort [12]. Higher post-recovery FEV1 was associated with a lower recurrence risk, indicating that impaired post-recovery pulmonary function was a marker of recurrence vulnerability [13]. This is clinically plausible because reduced airflow may reflect more extensive bullous destruction, small-airway disease, hyperinflation, and impaired ability to tolerate or heal a subsequent pleural leak. Interstitial pneumonia was also associated with unfavourable treatment outcomes and increased recurrence following surgery [14].

Disease-specific anatomical characteristics may provide further risk information. In Marfan syndrome, postoperative pneumothorax accumulated over prolonged follow-up, and chest deformity independently predicted recurrence, although the estimate was imprecise because of the small adult-inclusive cohort [15]. Mixed-population studies also identified older age, SSP status, smoking, and COPD as recurrence predictors [16]. Other reported associations included blebs or bullae, longer drainage duration, lower haemoglobin or haematocrit, and current smoking [17]. Cannabis exposure was associated with more severe bullous disease and more frequent postoperative recurrence in a surgical case-control cohort [18]. These mixed-cohort findings should be regarded as supportive rather than definitive SSP evidence, but they reinforce the importance of structural lung injury and potentially modifiable inhalational exposure.

The underlying disease may also influence the clinical course and the suitability of recurrence-prevention strategies. Nontuberculous mycobacterial pulmonary disease was associated with frequent recurrence and poor longer-term outcomes, although adjusted analyses primarily examined mortality rather than recurrence [19]. Old pulmonary tuberculosis prolonged drainage and increased management complexity in older patients but did not significantly affect recurrence-free survival [20]. This distinction is important because factors associated with a difficult initial admission may not necessarily predict a later pneumothorax.

For patients who are unsuitable for conventional surgery, less invasive pleural interventions may be considered. In SSP complicated by persistent air leak, a larger autologous blood-patch volume was associated with improved overall outcomes, although no adjusted recurrence-specific estimate was reported [21]. Late recurrence occurred in approximately 13% of patients in another blood-patch cohort [22], while blood-patch and chemical pleurodesis produced comparable recurrence outcomes in interstitial lung disease-associated SSP [23]. Non-intubated VATS with an integrated postoperative strategy was also feasible in a small high-risk cohort, although postoperative recurrence still occurred [24]. These studies suggest that treatment should be individualised according to surgical fitness, the underlying disease, anticipated survival, and the consequences of another episode.

The timing of previous recurrence may also modify the benefit of definitive intervention. Surgery prolonged the recurrence-free interval after an early recurrent episode but did not clearly provide the same benefit when the preceding recurrence-free interval exceeded one year [25]. Although this finding requires validation, an early recurrence may indicate that the original structural lesion or pleural abnormality remains untreated. It may therefore identify patients for whom definitive recurrence prevention has greater clinical value.

The surgical evidence suggests that effective lesion control and pleural intervention may be more important than the number of thoracoscopic ports. VATS with talc pleurodesis produced substantially lower recurrence than conventional treatment in a direct SSP comparison [26]. In a small SSP subgroup undergoing different pleural procedures, no recurrence occurred after total pleurectomy, whereas recurrence followed less extensive interventions; however, the small treatment groups prevent firm conclusions regarding superiority [27]. Uniportal and multiportal VATS produced identical crude recurrence rates, and port strategy was not independently associated with recurrence [28].

In a mixed surgical cohort, emphysema-related secondary pneumothorax predicted reoperation, while wedge resection was protective; however, the outcome combined postoperative complications and later recurrent pneumothorax [29]. A large national database reported lower recurrence following thoracotomy than VATS and identified thoracotomy and bullectomy as protective [30]. Nevertheless, PSP and SSP outcomes were not separable, and any recurrence advantage must be balanced against the greater procedural burden of thoracotomy, particularly in patients with limited cardiopulmonary reserve. The evidence should therefore not be interpreted as supporting routine thoracotomy for SSP.

Evidence in uncommon SSP populations remained limited. In apatinib-associated pneumothorax in patients with osteosarcoma, chest-tube drainage combined with staphylococcal enterotoxin C perfusion produced a numerically lower recurrence proportion than drainage alone, but the subgroup was small and the difference was not statistically significant [31]. Two-port and three-port VATS were also associated with similar long-term recurrence outcomes [32]. Overall, these findings support selecting a procedure according to the ability to control the responsible lesion and achieve an effective pleural intervention rather than according to port number alone.

Recurrence risk should be assessed using a multidimensional, phenotype-based approach rather than pneumothorax size alone. Assessment should include the underlying diagnosis, CT-defined emphysema, fibrosis or bullous disease, post-recovery pulmonary function, duration of air leak, previous recurrence pattern, physiological reserve, frailty, life expectancy, and patient preference. Patients with an air leak persisting beyond seven days should undergo early multidisciplinary review involving respiratory and thoracic surgical teams because prolonged air leak is associated with increased recurrence [6]. Smoking cessation should also be encouraged where relevant because smoking-related exposures were associated with recurrence in mixed spontaneous-pneumothorax cohorts [16-18].

For patients considered sufficiently fit, definitive recurrence prevention should be discussed during the index admission or following an early recurrent episode, particularly when severe structural lung disease, impaired pulmonary function, prolonged air leak, or non-COPD pathology is present [2,5-8,13,14,25,26]. Treatment may include VATS-based lesion control combined with an appropriate pleural procedure. In patients unsuitable for conventional surgery, medical pleurodesis or autologous blood-patch pleurodesis may be considered according to the cause of SSP, persistence of the air leak, local expertise, and the anticipated consequences of another episode [6,21-23]. Evidence favouring thoracotomy over VATS was indirect and should not be used to recommend routine thoracotomy for SSP [30].

These recommendations should support shared decision-making rather than be applied as a formal numerical prediction score. The included studies varied substantially in populations, recurrence definitions, follow-up periods, and outcomes; therefore, treatment decisions should balance the expected reduction in recurrence against procedural risk and the individual patient’s clinical circumstances.

The heterogeneity of the included evidence also explains why meta-analysis was not appropriate. Combining hazard ratios, odds ratios, crude recurrence proportions, readmission outcomes, and composite reoperation outcomes across different SSP phenotypes could have generated a statistically precise but clinically misleading estimate. The current evidence is more appropriately used to support qualitative, phenotype-based risk assessment.

Future studies should prospectively define SSP according to underlying disease and report ipsilateral and contralateral recurrence separately. Standardised outcomes at 30 days, 90 days, one year, and two years would improve comparability. Studies should also distinguish true recurrence following complete resolution from persistent or incompletely resolved pneumothorax. Multicentre cohorts are especially needed for uncommon phenotypes, including heritable disorders, malignancy-associated pneumothorax, and fibrotic lung disease. Adjustment should include structural disease severity, pulmonary function, smoking exposure, air-leak duration, treatment selection, frailty, and competing mortality. These data may ultimately permit the development of a validated SSP-specific prediction model.

Limitations 

This review searched only PubMed/MEDLINE, creating a risk of database-selection bias. Nineteen of the 50 reports sought for full-text assessment could not be retrieved despite searches of PubMed and PubMed Central, DOI and publisher websites, NHS or university library access, and attempts to contact corresponding authors where possible. Their absence creates a risk of availability bias because some may have met the eligibility criteria and could have identified additional predictors, null associations, or treatment outcomes. Consequently, the direction, magnitude, and certainty of some conclusions may have differed had these reports been available. The missing reports were therefore transparently recorded as not retrieved rather than excluded on scientific grounds. The review protocol was not prospectively registered with PROSPERO or another systematic-review registry, which may increase the risk of selective methodological or outcome reporting, although the eligibility criteria and review methods have been reported transparently. The included evidence was predominantly retrospective and was subject to treatment-selection bias, referral bias, competing mortality, unequal follow-up, and heterogeneous definitions of SSP and recurrence. The eligibility criteria permitted adult-inclusive disease cohorts and mixed spontaneous-pneumothorax studies when they evaluated a relevant recurrence determinant; although this increased the breadth of evidence, it also introduced indirectness. Several studies reported recurrence as a secondary outcome or used composite readmission or reoperation outcomes, and adjusted recurrence-specific estimates were frequently unavailable. Consequently, this review supports qualitative, phenotype-based risk stratification rather than a pooled recurrence rate or a validated numerical prediction model.

## Conclusions

Recurrence after SSP is influenced by the underlying disease phenotype, severity of structural lung damage, physiological reserve, behaviour of the index episode, and whether effective recurrence prophylaxis is performed. The most consistent risk indicators were severe emphysematous or fibrotic disease, non-COPD aetiology, prolonged air leak, impaired pulmonary function, early previous recurrence, and selected disease-specific anatomical or radiographic features.

Definitive recurrence-prevention strategies, including VATS-based treatment, pleural procedures, medical pleurodesis, and selected less invasive approaches, generally produced lower recurrence than drainage alone. However, no single intervention is appropriate for every patient. Treatment should balance the anticipated reduction in recurrence against operative fitness, procedural burden, underlying disease trajectory, life expectancy, and patient preference. SSP should therefore be managed using an individualised, phenotype-based assessment incorporating CT findings, pulmonary function, air-leak duration, recurrence timing, and suitability for definitive pleural intervention. Prospective multicentre studies should standardise recurrence definitions, distinguish ipsilateral from contralateral events, account for competing mortality, and develop externally validated SSP-specific prediction tools.

## Appendices

Appendix 1

**Table 3:** Complete PubMed/MEDLINE search strategy COPD, chronic obstructive pulmonary disease; SSP, secondary spontaneous pneumothorax; VATS, video-assisted thoracoscopic surgery; tiab, title/abstract; MeSH, Medical Subject Headings

Search information	Details
Database	PubMed/MEDLINE
Search date	19 July 2026
Date restriction	No date restriction
Filters applied	English language; humans; adults aged 19 years or older
Records retrieved	77
Complete search strategy	("secondary spontaneous pneumothorax"[tiab] OR SSP[tiab] OR (pneumothorax[tiab] AND (secondary[tiab] OR "underlying lung disease"[tiab] OR "chronic lung disease"[tiab] OR COPD[tiab] OR "chronic obstructive pulmonary disease"[tiab] OR emphysema[tiab] OR "interstitial lung disease"[tiab] OR interstitial[tiab] OR fibrosis[tiab] OR malignan*[tiab] OR cancer[tiab]))) AND (recurren*[tiab] OR recurrent[tiab] OR relapse[tiab] OR "recurrence-free"[tiab]) AND (predict*[tiab] OR prognos*[tiab] OR "risk factor*"[tiab] OR determinant*[tiab] OR associated[tiab] OR association[tiab] OR pleurodesis[tiab] OR surgery[tiab] OR surgical[tiab] OR thoracoscop*[tiab] OR VATS[tiab] OR bullectom*[tiab] OR "persistent air leak"[tiab] OR "Air Leaks"[Mesh]) NOT (traumatic[tiab] OR iatrogenic[tiab] OR catheter*[tiab] OR biopsy[tiab] OR ventilat*[tiab] OR neonatal[tiab] OR newborn*[tiab] OR infant*[tiab] OR child*[tiab] OR paediatric[tiab] OR pediatric[tiab] OR catamenial[tiab])

Appendix 2

**Table 4:** Availability accounting Availability of reports sought for full-text retrieval after title and abstract screening. “Include” indicates records considered likely eligible at screening, whereas “Maybe” indicates records for which eligibility remained uncertain pending full-text assessment. Reports not retrieved were unavailable to the review team and were not excluded on scientific grounds.

Title/abstract category	Reports sought	Retrieved	Not retrieved
Include	34	23	11
Maybe	16	8	8
Total	50	31	19

Appendix 3

**Table 5:** Reports excluded after full-text assessment Reports excluded after full-text assessment and the primary reason for exclusion. Each report was assigned one reason based on the prespecified eligibility criteria. Reports that could not be retrieved were not included in this table and were recorded separately as “reports not retrieved.”

Report	Reason for exclusion
Ochi T, Kurihara M, Tsuboshima K, Nonaka Y, Kumasaka T. Dynamics of thoracic endometriosis in the pleural cavity. PLoS One. 2022;17:e0268299. doi:10.1371/journal.pone.0268299	Postoperative recurrence was reported, but the study did not evaluate an eligible recurrence-specific predictor or comparator.
Hong PY, Cai JH, Yan YB, et al. Clinical characteristics and in-hospital outcomes of pneumothorax in pneumoconiosis: a tertiary referral cohort study. Front Med (Lausanne). 2026;13:1834578. doi:10.3389/fmed.2026.1834578	Recurrent pneumothorax was used as a baseline subgroup; the study did not evaluate a post-index recurrence outcome or a determinant of subsequent recurrence.
